# Paraspeckles modulate the intranuclear distribution of paraspeckle-associated *Ctn RNA*

**DOI:** 10.1038/srep34043

**Published:** 2016-09-26

**Authors:** Aparna Anantharaman, Mahdieh Jadaliha, Vidisha Tripathi, Shinichi Nakagawa, Tetsuro Hirose, Michael F. Jantsch, Supriya G. Prasanth, Kannanganattu V. Prasanth

**Affiliations:** 1 Department of Cell and Developmental Biology, University of Illinois at Urbana-Champaign, 601 S Goodwin Avenue, Urbana, IL 61801, USA; 2 RNA Biology Laboratory, Faculty of Pharmaceutical Sciences, Hokkaido University, Kita 12-jo Nishi 6-chome, Kita-ku, Sapporo 060-0812, Japan; 3 Institute of Genetic Medicine, Hokkaido University, Sapporo 060-0815, Japan; 4 Department of Cell and Developmental Biology, Center of Anatomy and Cell Biology, Medical University of Vienna, Schwarzspanierstrasse 17, A-1090, Vienna, Austria

## Abstract

Paraspeckles are sub-nuclear domains that are nucleated by long noncoding RNA *Neat1*. While interaction of protein components of paraspeckles and *Neat1* is understood, there is limited information on the interaction of non-structural RNA components with paraspeckles. Here, by varying paraspeckle number and size, we investigate how paraspeckles influence the nuclear organization of their non-structural RNA component *Ctn RNA*. Our results show that *Ctn RNA* remains nuclear-retained in the absence of intact paraspeckles, suggesting that they do not regulate nuclear retention of *Ctn RNA*. In the absence of *Neat1*, *Ctn RNA* continues to interact with paraspeckle protein NonO to form residual nuclear foci. In addition, in the absence of *Neat1*-nucleated paraspeckles, a subset of *Ctn RNA* localizes to the perinucleolar regions. Concomitant with increase in number of paraspeckles, transcriptional reactivation resulted in increased number of paraspeckle-localized *Ctn RNA* foci. Similar to *Neat1*, proteasome inhibition altered the localization of *Ctn RNA*, where it formed enlarged paraspeckle-like foci. Super-resolution structured illumination microscopic analyses revealed that in paraspeckles, *Ctn RNA* partially co-localized with *Neat1*, and displayed a more heterogeneous intra-paraspeckle localization. Collectively, these results show that while paraspeckles do not influence nuclear retention of *Ctn RNA*, they modulate its intranuclear compartmentalization.

The eukaryotic nucleus is the site where crucial cellular events such as DNA replication, RNA synthesis and processing take place^1^. To facilitate the efficient co-ordination of these pathways, the nucleus is further compartmentalized into sub-nuclear domains such as nuclear speckles, Cajal bodies and nucleoli^1^,^2^,^3^,^4^,^5^. These subnuclear domains are known to regulate several important cellular processes such as ribosomal RNA transcription and pre-mRNA splicing^3^,^4^. Recent studies have shown that nuclear bodies are not random aggregates of proteins or RNAs, but are steady-state structures that are formed by dynamic interactions of protein-protein and/or protein-RNA components^1^,^4^,^6^. The interactions between protein and RNA components of nuclear domains have been investigated in-depth for some nuclear domains, but remain uncharacterized for others. A comprehensive understanding of these interactions will provide insights into the biogenesis, maintenance and function of sub-nuclear domains.

Paraspeckles are sub-nuclear bodies that are detected as variable number of discrete dots, and are preferentially located adjacent to nuclear speckles^7^,^8^. Paraspeckles contain ribonucleoprotein complexes that are formed around *NEAT1 (Nuclear Enriched Abundant Transcript 1*/*MENε*/*β* long noncoding RNA^4^,^9^,^10^,^11^,^12^,^13^,^14^. Two non-coding *Neat1* RNA isoforms are transcribed from the same promoter through alternative 3′end processing^8^,^9^,^10^,^12^,^14^,^15^. The mouse *Men ε* (*Neat1_v1*) is a 3.17-kb long poly(A) RNA, while *Men β* (*Neat1_v2*) is a 20.7-kb long transcript that harbors a triple helical structure in its 3′end^12^,^14^,^16^,^17^. Apart from *Neat1*, paraspeckles contain several paraspeckle-localized RNA-binding proteins, collectively termed as PSPs (paraspeckle-associated proteins). In addition to the three core PSPs that are members of the Drosophila Behavior Human Splicing (DBHS) family - NonO, SFPQ and PSP1, paraspeckles also contain an additional ~40 or more PSPs^7^,^8^,^15^. The protein components of paraspeckles are known to participate in several RNA metabolic pathways, including pre-mRNA processing and RNA stability.

Previous studies demonstrated *Neat1* as the organizational component of paraspeckles^4^,^9^,^10^,^12^,^13^,^14^. *NEAT1_v2* forms the paraspeckle core, whereas *NEAT1_v1* is recruited as a subsidiary factor^12^,^18^. The depletion of *Neat1* has been shown to disrupt paraspeckle structure^9^,^10^,^12^,^14^. Studies using a LacI reporter live cell imaging system to visualize the inducible transcription of *Neat1* and paraspeckle proteins demonstrate that active transcription of *Neat1* regulates paraspeckle maintenance^19^. This is supported by other studies showing the disruption of intact paraspeckles upon transcription inhibition, and reformation upon transcription reactivation^12^.

*Neat1* plays an important role in basic physiological functions and diseases^20^,^21^. Upon immune stimuli, *NEAT1* facilitates the relocation of splicing factor proline/glutamine-rich (SFPQ), a *NEAT1*-associated PSP, from the IL8 (Interleukine 8) promoter to paraspeckles, leading to transcriptional activation of IL8^22^. *NEAT1* is also known to repress transcription of several genes, including *ADARB2* (Adenosine deaminase that Acts on RNA 3), by sequestering the transcription repressor SFPQ from the promoters of these protein-coding genes^23^. Finally, *NEAT1*-enriched paraspeckles have been suggested to be involved in the nuclear retention of A-to-I edited transcripts^9^. *Neat1*-knockout mice are viable under laboratory growth conditions, and thus, paraspeckles are considered to be nonessential nuclear bodies that are formed upon certain environmental triggers such as viral infection, proteasome inhibition and differentiation^14^,^22^,^23^,^24^.

Members of the paraspeckle-resident PSPs interact with *Neat1* and influence the spatial arrangement of *Neat1* within paraspeckles. For example, NonO and SFPQ selectively associate with, and stabilize *NEAT1_v2*, thus contributing to the organization of the paraspeckle structure^12^. Although the interaction of PSPs and *Neat1* has been studied in detail, information regarding the organization and behavior of non-structural RNA components of paraspeckles is scant^25^,^26^. *Ctn RNA* is an 8 kb long, mouse-specific, nuclear-retained RNA that is induced as part of the antiviral response^26^. Apart from its homogenous distribution in the nucleoplasm, it also localizes to paraspeckles. *Ctn RNA* regulates the expression of its protein-coding partner, *Cat2* (mouse cationic amino acid transporter 2)^26^. mCAT2 facilitates the cellular uptake of L-arginine, which is utilized as a substrate for the synthesis of nitric oxide (NO) in the cell. Both *Ctn RNA* and *mCat2* mRNA are encoded by the *Slc7a2* gene, however, due to alternative poly(A) site selection, *Ctn RNA* contains a longer unique 3′UTR^26^. The long 3′UTR of *Ctn RNA* contains several inverted repeats of SINE origin, and several of the adenosines within these repeats undergo Adenosine-to-Inosine (A-to-I) editing by ADAR family of cellular enzymes^26^. Upon cellular stress, *Ctn RNA* is cleaved at the 3′UTR and is exported into the cytoplasm where it is translated to form mCAT2 protein^26^. Knockdown of *Ctn RNA* does not affect paraspeckle integrity, suggesting that it is a non-structural RNA component of paraspeckles^10^,^26^. *Ctn RNA* has been shown to interact with PSPs – NonO and PSP1^26^. However, apart from this limited information, not much is known about the interaction of *Ctn RNA* with paraspeckles. In this study, we investigated how alteration in paraspeckle number and size affects the association of *Ctn RNA* with paraspeckles. In addition, by utilizing *Ctn RNA* as a model system, we determined the potential involvement of A-to-I editing in the nuclear retention and paraspeckle association of RNA.

## Results

### *Ctn RNA* is nuclear-retained in the absence of intact paraspeckles, and forms ‘residual’ paraspeckle foci

**Figure 1:**
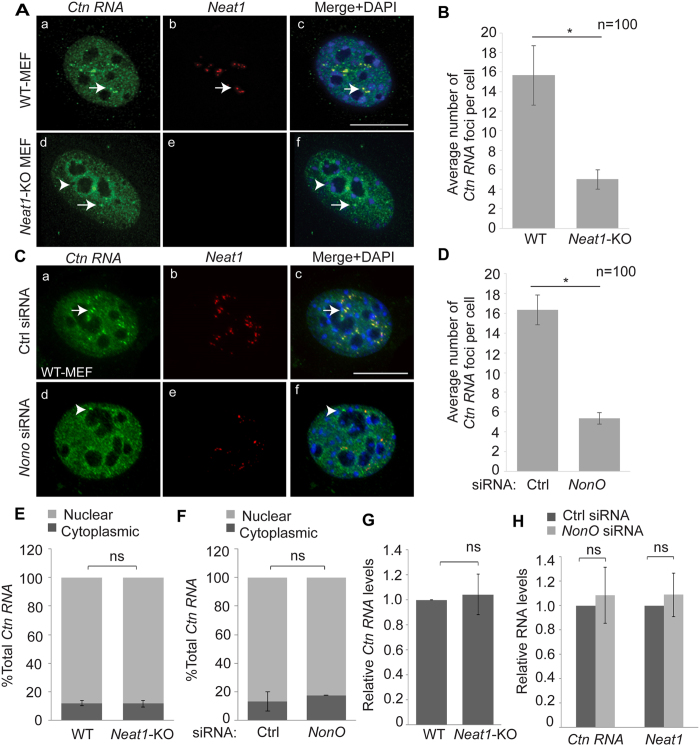
*Ctn RNA* is nuclear-retained in absence of intact paraspeckles. (**A**) RNA-FISH to detect *Ctn RNA* (green) and *Neat1* (red) in DRB-recovered (3 hrs) WT and *Neat1*-KO MEFs. Arrow (a–f) indicates *Ctn RNA* foci and arrowhead (d,f) indicates perinucleolar localization of *Ctn RNA*. (**B**) Graph showing average number of *Ctn RNA* foci per cell in WT and *Neat1*-KO MEFs. (**C**) RNA-FISH to detect *Ctn RNA* and *Neat1* in DRB-recovered control (Ctrl) and *NonO*-depleted transformed WT-MEFs. Please note that *Ctn RNA* shows increased paraspeckle association upon DRB recovery (please see Fig. 4). Arrow (a,c) marks *Ctn RNA* and *Neat1* positive paraspeckle. Arrowhead (d,f) shows *Ctn RNA* positive but *Neat1* negative paraspeckle-like nuclear body. (**D**) Graph showing average number of *Ctn RNA* foci per cell in (Ctrl) and *NonO*-depleted transformed WT-MEFs. (**E,F**) RT-qPCR to estimate *Ctn RNA* levels in nuclear and cytoplasmic fractions of (**E**) WT and *Neat1*-KO MEFs and (**F**) Ctrl and *NonO*-depleted MEFs. (**G**) RT-qPCR to detect total levels of *Ctn RNA* in WT and *Neat1*-KO MEFs. (**H**) Total levels of *Ctn RNA* in control and *NonO* siRNA treated MEFs. 3′UTR-1 primer pair has been used to measure *Ctn RNA* levels (Figure S2). *Gapdh* was used as the normalization control in RT-qPCR experiments. Scale bar indicates 10 μm. Error bars in (**B,D,E–H**) represent mean ± SD of three independent experiments. *P < 0.05, ns: not significant, using Student’s t test.

Previous studies have speculated that paraspeckles are involved in the nuclear retention of A-to-I edited transcripts^9^,^10^,^26^,^27^,^28^,^29^. *Ctn RNA* is a paraspeckle-associated transcript that is A-to-I edited within its long 3′UTR^26^. To investigate if paraspeckles regulate the nuclear retention of *Ctn RNA*, we determined the cellular localization of *Ctn RNA* in WT-MEFs (Mouse embryonic fibroblasts) and *Neat1*-KO (knockout) MEFs by RNA-FISH (RNA-Fluorescent *in situ* hybridization) analysis^24^. *Neat1* lncRNA has been shown to nucleate paraspeckles and thus, in the absence of *Neat1*, paraspeckle structure is disrupted^9^,^10^,^12^,^14^,^19^. We observed that in WT-MEFs, *Ctn RNA* co-localized with *Neat1* with in the intact paraspeckles. In addition, *Ctn RNA* also displayed homogenous nuclear distribution (Fig. 1Aa–c and S1A–C). In *Neat1*-KO MEFs too, where intact paraspeckles were absent, *Ctn RNA* continued to localize in the nucleus (Figs 1Ad–f and S1C). Since paraspeckle protein NonO has been shown to interact with, and influence the nuclear localization of hyper-edited RNAs, we ascertained if NonO regulates nuclear retention of A-to-I edited *Ctn RNA*^9^. We performed RNA-FISH to determine *Ctn RNA* and *Neat1* co-localization in control and *NonO*-depleted WT-MEFs (Figs 1Ca–f, and S1D–E). Paraspeckle proteins NonO and SFPQ associate with, and stabilize the longer isoform of *Neat1*, thus, stabilizing paraspeckle structure^12^. *Neat1* RNA-FISH analysis confirmed the reduction in paraspeckle number in NonO-depleted cells (Figs 1C and S1D,E). However, NonO-depleted cells continued to show nuclear and paraspeckle association of *Ctn RNA*, suggesting that NonO does not influence the nuclear retention of *Ctn RNA* (Fig. 1C).

Next, we determined the total levels of *Ctn RNA* in nuclear cytoplasmic fractions by RT-qPCR (Reverse transcription quantitative PCR) analysis. In order to measure *Ctn RNA* levels specifically, and not *mCat2*, we used a primer pair that is unique to the long 3′UTR of *Ctn RNA* (Fig. S2A). In agreement with RNA-FISH analysis, we observed predominantly nuclear enrichment of *Ctn RNA* in the presence or absence of *Neat1* or NonO (Figs 1E,F and S2B–E). We also investigated if the disruption of paraspeckle resulted in any changes in the relative abundance of *Ctn RNA*. To this end, we measured total *Ctn RNA* levels in WT and *Neat1*-KO MEFs or control and *NonO*-depleted MEFs. We did not observe any change in the total levels of *Ctn RNA* in the absence of *Neat1* or NonO (Fig. 1G,H). Together, these results show that paraspeckles do not affect the nuclear retention or cellular abundance of *Ctn RNA*.

In WT-MEFs, *Ctn RNA* is localized to paraspeckles, as confirmed by co-staining of cells with *Neat1* (Fig. 1Aa–c). However, there was a dramatic reduction in the number of intranuclear *Ctn RNA* foci in the *Neat1*-KO cells (Fig. 1Ad–f,B). We observed a few, but prominent *Ctn RNA* positive intranuclear ‘residual’ foci in *Neat1*-KO MEFs (3–4/nucleus in KO cells instead of 15–20 paraspeckles/nucleus in WT cells) (Fig. 1A,B). In the absence of *Neat1*, a subset of *Ctn RNA* localized to the perinucleolar space (Fig. 1A). On the other hand, in NonO-depleted cells, we observed that a few less prominent residual *Ctn RNA* foci continued to persist, some of which did not co-localize with *Neat1* (Fig. 1Cd–f,D, see arrowhead). Collectively, these results suggest that in absence of intact paraspeckle structure, *Ctn RNA* foci form fewer residual nuclear foci, and also localize to the perinucleolar compartment.

### *Ctn RNA* associates with other paraspeckle components in absence of *Neat1*

**Figure 2:**
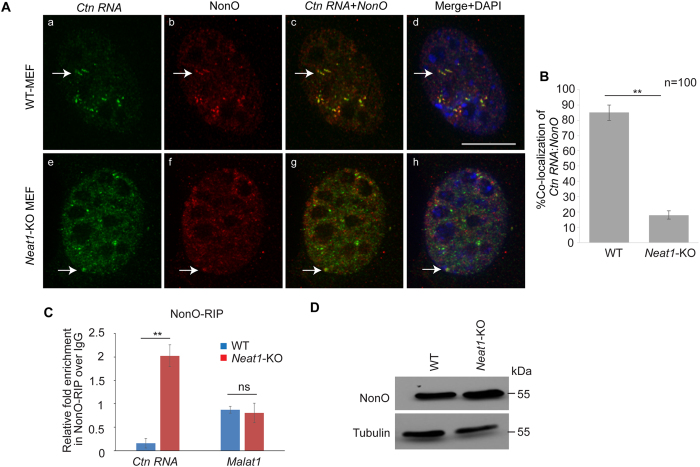
*Ctn RNA* interacts with paraspeckle component NonO in absence of intact paraspeckles. (**A**) RNA-FISH to detect *Ctn RNA* (green) and NonO (red) in DRB-recovered WT and *Neat1*-KO MEFs. Scale bar indicates 10 μm. Arrow (a–h) indicates *Ctn RNA* and NonO positive nuclear foci. DNA is counterstained with DAPI (blue). (**B**) Graph showing percentage co-localization in WT and *Neat1*-KO MEFs. (**C**) NonO-RIP (RNA immunoprecipitation) followed by RT-qPCR analysis of *Ctn RNA* to determine interaction of NonO and *Ctn RNA* in WT and *Neat1*-KO MEFs. (**D**) Western blot showing NonO levels in WT and *Neat1*-KO MEFs. Tubulin was used as a loading control. *Gapdh* was used as the normalization control in RT-qPCR experiments. Error bars in (**B,C**) represent mean ± SD of three independent experiments. **P < 0.01 using Student’s t test.

Next, we wanted to determine if disruption of paraspeckle structure affects the interaction of *Ctn RNA* with other paraspeckle components. *Ctn RNA* has been shown to interact with paraspeckle proteins such as NonO and PSP1^26^. In the current study, we ascertained if the *Ctn RNA* continues to associate with paraspeckle proteins in the absence of *Neat1*. To this end, we determined the localization of *Ctn RNA* and paraspeckle protein NonO by performing RNA-FISH followed by immunostaining for NonO in WT and *Neat1*-KO MEFs. We observed that as shown in previous studies, *Ctn RNA* and NonO co-localized in WT-MEFs (Fig. 2Aa–d)^26^. Surprisingly, in the *Neat1* KO cells, a few but not all of the bright *Ctn RNA* foci co-localized with NonO-stained foci (Fig. 2Ae–h,B; please see the arrows).

However, due to the reduction in the number of *Ctn RNA* and NonO foci in the *Neat1* KO cells, it was not possible to determine if the efficiency of this interaction was same as seen in case of WT-MEFs. Therefore, to quantitate the interaction between *Ctn RNA* and NonO, we performed ribonucleoprotein immunoprecipitation (RIP) studies using the NonO antibody followed by RT-qPCR. We observed that NonO displayed increased interaction with *Ctn RNA* in *Neat1*-KO cells but not with *Malat1*, another highly abundant nuclear-retained long noncoding RNA (Fig. 2C). However, we also observed that the total levels of NonO were marginally increased in *Neat1*-KO MEFs (Fig. 2D). Thus, it is possible that the increased interaction between *Ctn RNA* and NonO in *Neat1*-KO MEFs, is due to the higher levels of NonO in the *Neat1*-KO MEFs. Collectively, these studies conclude that *Ctn RNA* is able to interact with other paraspeckle components in the absence of *Neat1* or an intact paraspeckle structure.

### ADARs do not regulate the association of *Ctn RNA* to paraspeckles

**Figure 3:**
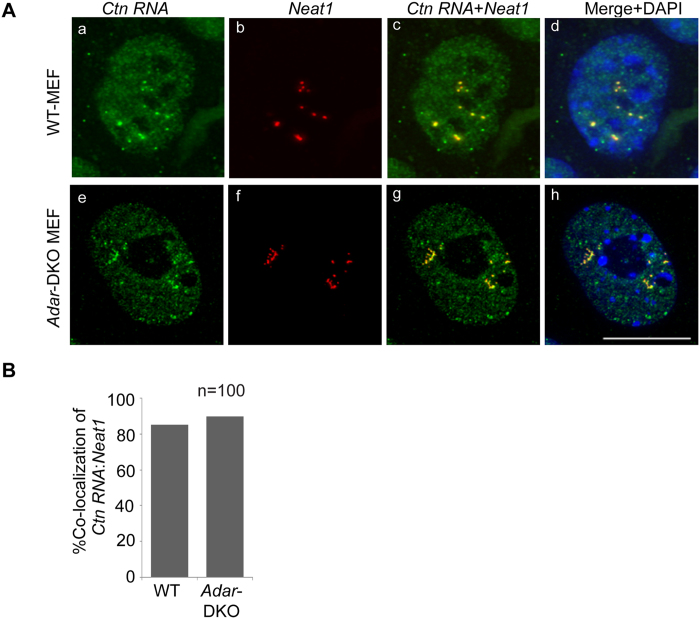
ADARs do not influence the association of A-to-I edited *Ctn RNA* to paraspeckles. (**A**) RNA-FISH to detect *Ctn RNA* (green) and *Neat1* (red) in DRB-recovered WT and *Adar1*/*Adar2* double knockout-KO (DKO) MEFs. DNA is counterstained with DAPI (blue). Scale bar indicates 10 μm. (**B**) % co-localization of *Ctn RNA* and *Neat1* in the paraspeckles of DRB-recovered WT and *Adar1/Adar2* double knockout-KO (DKO) MEFs.

There has been an ongoing debate about the potential involvement of A-to-I editing in the retention of hyper-edited transcripts to nucleus and also to paraspeckles^9^,^26^,^29^,^30^,^31^,^32^. While some earlier studies have shown that inverted Alu (IRAlu) containing RNA is retained in the nucleus by paraspeckles, other studies show that IRAlu containing RNA are efficiently exported into the cytoplasm^9^,^26^,^29^,^30^,^31^,^32^. A very recent study reported that in LPS-induced immune cells, a significant fraction of transcripts with hyper-edited regions tends to be retained in the nucleus^33^. In its 3′UTR, *Ctn RNA* harbors three inverted repeats (IR) of SINE origin that are inverted with respect to the forward repeat (FwR) (Fig. S2A)^26^. Double stranded regions formed by pairing of FwR and IR are frequently A-to-I edited by RNA editing enzyme Adenosine deaminases acting on RNA (ADARs)^34^. While three forms of this enzyme exist in human cells -ADAR1, 2 and 3, only ADAR1 and ADAR2 display editing activity^34^. In case of *Ctn RNA*, we have previously reported that the several adenosines within the FwR and IR2 repeats undergo A-to-I editing^26^. Taken together with the fact that the 3′UTR was involved in the nuclear localization of *Ctn RNA*, it was hypothesized that editing of *Ctn RNA* and its association with paraspeckle components such as NonO could influence it nuclear retention. Thus, we ascertained if A-to-I editing regulates the paraspeckle association and nuclear retention of *Ctn RNA*. We examined the cellular localization of *Ctn RNA* in WT and *Adar1/Adar2* double knockout MEFs by RNA-fluorescence *in situ* hybridization (RNA-FISH) (Fig. 3A)^35^,^36^. In WT-MEFs, *Ctn RNA* showed homogenous nuclear distribution, and also localized to paraspeckles, as confirmed by co-staining of cells with *Neat1* (Fig. 3Aa–d). *Ctn RNA* displayed similar paraspeckle localization in the *Adar*-KO MEFs (Fig. 3A,B), even though these cells showed complete loss of A-to-I editing within the FwR and IR2 repeats of *Ctn RNA* (data not shown). Furthermore, *Ctn RNA* showed similar paraspeckle localization both control and *Adar1*-depleted (using siRNAs) transformed WT-MEFs (Fig. S3Aa–h,B). Together, our results indicate that in MEFs, A-to-I editing does not influence the nuclear localization and paraspeckle-association of *Ctn RNA*.

### *Ctn RNA* foci increase in number upon transcription reactivation

**Figure 4:**
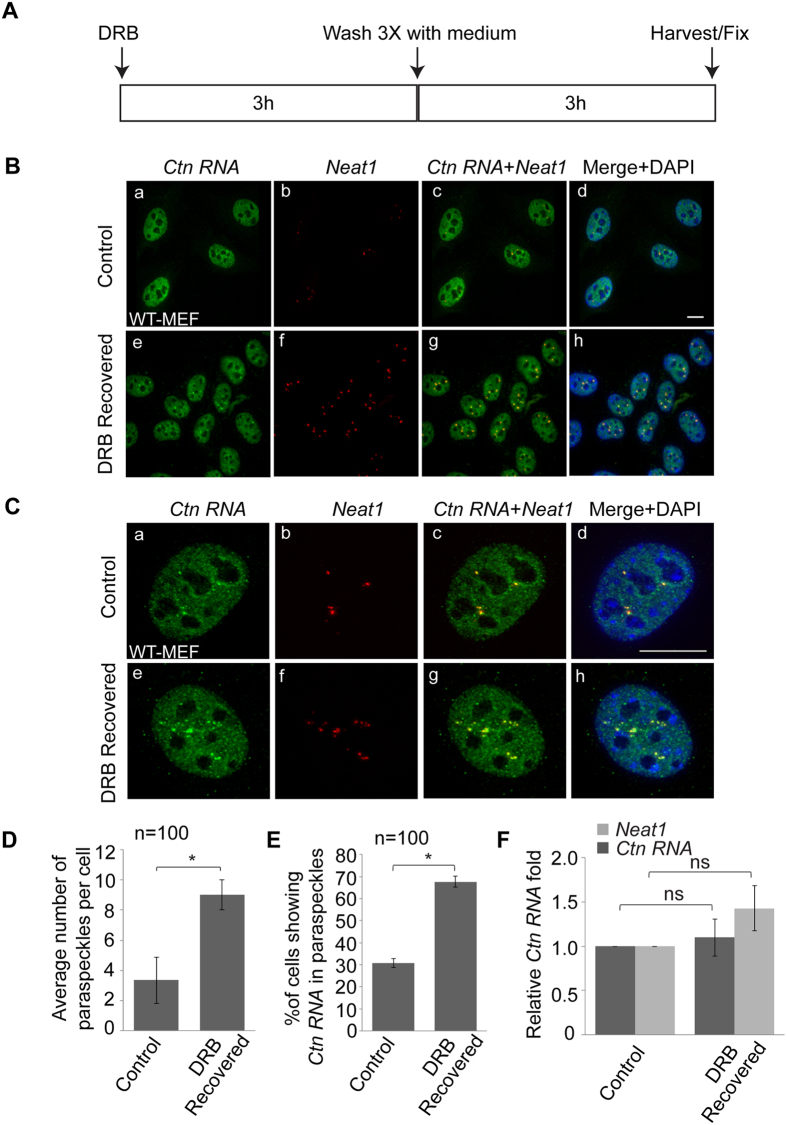
Number of *Ctn RNA* positive foci and their association with paraspeckles is increased upon transcriptional reactivation. (**A**) Schematic showing the experimental design. (**B**) RNA-FISH to detect *Ctn RNA* (green) and *Neat1* (red) in control (ethanol) and DRB-recovered transformed WT-MEFs. (**C**) RNA-FISH analysis of *Ctn RNA* and *Neat1* localization in a single cell of control (ethanol-treated) and DRB-recovered transformed WT-MEF. (**D**) Graph showing average number of paraspeckles per cell in control (ethanol-treated) and DRB-recovered transformed WT-MEFs. (**E**) Graph showing percentage of cells positive for *Ctn RNA* foci in control (ethanol-treated) and DRB-recovered transformed WT-MEFs. (**F**) RT-qPCR analysis of *Ctn RNA* levels in control (ethanol-treated) and DRB-recovered transformed WT-MEFs. *Gapdh* was used as the normalization control in RT-qPCR experiments. Scale bar indicates 10 μm. DNA is counterstained with DAPI (blue). Error bars in (**D–F**) represent mean ± SD of three independent experiments. *P < 0.05, ns: not significant using Student’s t test.

The adenosine analogue 5,6-dichloro-1-β-D-ribofuranosylbenzimidazole (DRB) inhibits RNA polymerase II-mediated transcriptional elongation by inactivating the activity of pTEFb kinase. Previous studies have shown that paraspeckle protein PSP1 re-localized to perinucleolar caps after 5 min of DRB treatment and remarkably, paraspeckles structure was disrupted ~40 min after DRB treatment^12^,^19^. Interestingly, paraspeckles reformed upon removal of DRB from the culture medium indicating that paraspeckle maintenance is coupled with *Neat1* transcription^12^,^19^. We have previously reported that *Ctn RNA* foci were also disrupted upon RNA pol II transcription inhibition, including treatment of cells with DRB^26^. Therefore, we investigated the reassembly of *Ctn RNA* foci upon transcription reactivation. To this end, we treated transformed WT-MEFs with DRB for three hours. Next, we removed DRB by washing cells with medium, cultured the cells in fresh media for another 3 hours, performed co-RNA-FISH and counted the number of *Ctn RNA* foci in both untreated and DRB recovered cells (Fig. 4A). In agreement with previous studies that demonstrated increased paraspeckle number upon DRB recovery, we observed a two to three-fold increase in the number of paraspeckles/cell, as observed by *Neat1* staining (~4/cell in control to ~8/cell in treated) (Fig. 4B–D)^19^. In addition, we also observed a concomitant two-fold increase in number of cells with *Ctn RNA* decorating *Neat1* positive paraspeckles (Fig. 4B,C,E) (~30% of cells showed *Ctn RNA* foci in control cells whereas ~70% of transcription-reactivated cells showed *Ctn RNA* positive paraspeckles). Furthermore, *Ctn RNA* staining within the paraspeckles appeared more prominent in transcription-reactivated cells, indicating a possible increase in the number of *Ctn RNA* molecules per paraspeckle during DRB-recovery.

Previous studies have shown that while *Neat1* is a highly unstable RNA (half-life ~2 h), *Ctn RNA* constitutes a stable pool of RNA (half-life ~8 h)^26^,^37^. We wanted to determine if the increased paraspeckle number and *Ctn RNA* association with paraspeckles during transcription reactivation was due to changes in the total levels of *Neat1* and *Ctn RNA*. To this end, we measured the total levels of *Neat1* and *Ctn RNA* in control and DRB-recovered transformed WT-MEFs by RT-qPCR (Fig. 4F). The results showed that neither *Neat1* nor *Ctn RNA* displayed any significant changes in abundance upon DRB recovery (Fig. 4F). Together, these results demonstrate that *Ctn RNA* shows increased localization to paraspeckles upon transcriptional reactivation without a corresponding increase in total levels of *Neat1* or *Ctn RNA*.

### *Ctn RNA* forms enlarged foci upon proteasome inhibition

**Figure 5:**
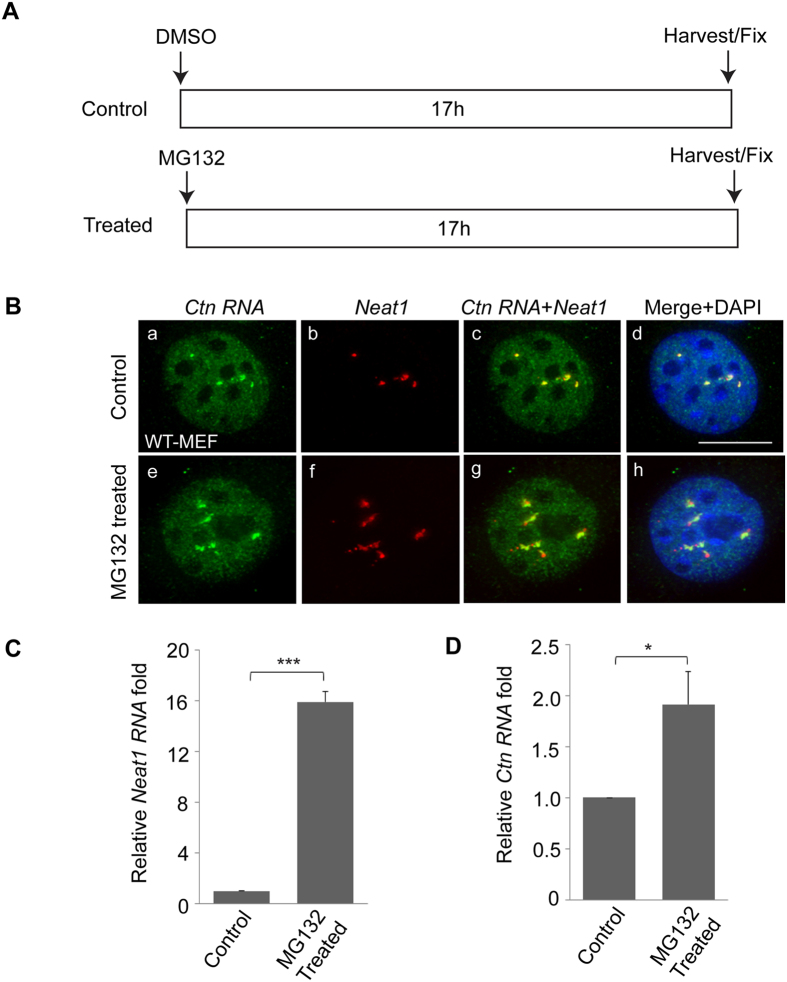
*Ctn RNA* forms enlarged foci in proteasome-inhibited cells. (**A**) Schematic showing the experimental design. (**B**) RNA-FISH analysis of *Ctn RNA* (green) and *Neat1* (red) localization in control (DMSO-treated) and MG132-treated transformed WT-MEFs. Scale bar indicates 10 μm. DNA is counterstained with DAPI (blue). (**C**) RT-qPCR analysis of *Neat1* RNA levels in control (DMSO-treated) and MG132-treated transformed WT-MEFs. (**D**) RT-qPCR analysis of *Ctn RNA* levels in control (DMSO-treated) and MG132-treated transformed WT-MEFs. *Gapdh* was used as the normalization control in RT-qPCR experiments. Error bars in (**C,D**) represent mean ± SD of three independent experiments. ***P < 0.001, *P < 0.05 using Student’s t test.

Recently, it was demonstrated that paraspeckles become dramatically enlarged upon proteasome inhibition^23^. Surprisingly, this enlargement in paraspeckle size was shown to be a result of *Neat1* transcription activation, and not because of the accumulation of undegraded PSPs. In fact, upon proteasome inhibition, PSPs were sequestered into paraspeckles as evidenced by 50% depletion of these proteins from the nucleoplasm^23^. To determine if paraspeckle RNA component, *Ctn RNA* showed any changes upon proteasome inhibition, we treated transformed WT-MEFs with the proteasome inhibitor MG132 for 17 h and compared the *Ctn RNA* foci with control (DMSO-treated) cells (Fig. 5A). We observed that *Ctn RNA* also formed enlarged nuclear foci upon MG132-treatment (Figs 5B and S4).

We wondered whether proteasome-inhibition also altered transcription from *Ctn RNA* locus. To test this aspect, we measured *Ctn RNA* levels in DMSO and MG132-treated transformed WT-MEFs by RT-qPCR analysis (Fig. 5C,D). Consistent with a previous report, we observed significant increase in the levels of *Neat1* in proteasome-inhibited cells (Fig. 5C)^23^. On the other hand, *Ctn RNA* showed only a small but significant increase in total RNA levels (Fig. 5D; ~2 fold of increase of *Ctn RNA* compared to ~16 fold of *Neat1*). Collectively, these results showed that upon proteasome inhibition, *Ctn RNA* formed enlarged foci. In addition, the *Ctn RNA* levels were only moderately increased in comparison to the marked increase in *Neat1* levels.

**Figure 6:**
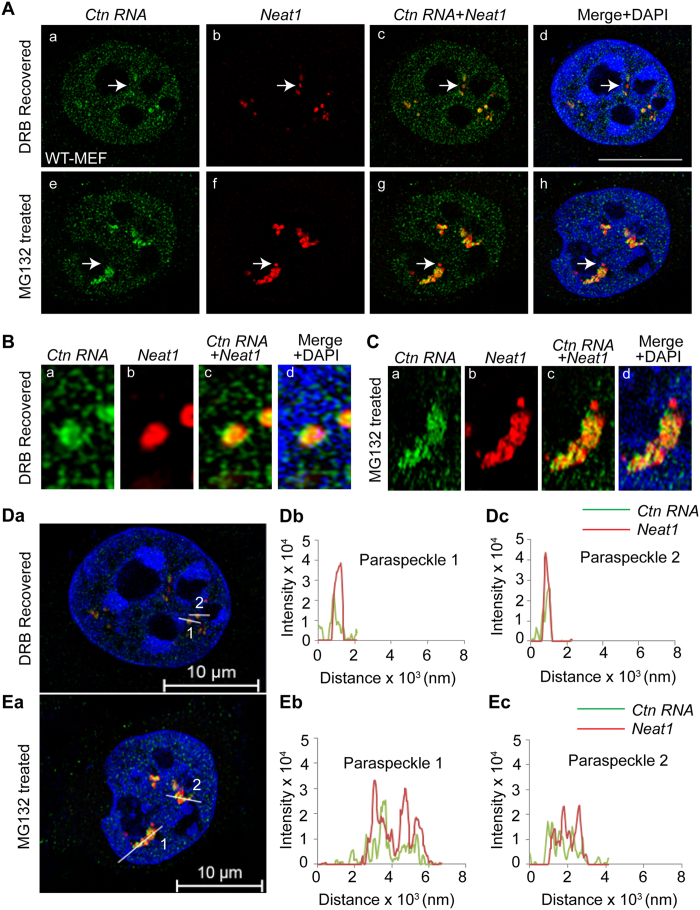
Intra-paraspeckle localization of *Ctn RNA*. (**A**) Super-resolution structured illumination microscopy (SR-SIM) of *Ctn RNA* (green) and *Neat1* (red) localization in DRB recovered and MG132-treated transformed WT-MEFs. Scale bar indicates 10 μm. Arrow (a–h) indicates paraspeckle where *Ctn RNA* does not show co-localization with *Neat1*. (**B,C**) Co-localization of a single paraspeckle in (**B**) DRB recovered and (**C**) MG132-treated transformed WT-MEF. (**D,E**) Quantitation of *Ctn RNA* and *Neat1* co-localization in (Da–c) DRB recovered and (Ea–c) MG132 treated transformed WT-MEFs (performed using ZEN 2012). Numbers in image indicate the specific paraspeckle analyzed and corresponds to the number mentioned in the graph. For example, “1” in image refers to “paraspeckle 1” in graph.

*Ctn RNA* and *Neat1* localization studies, especially in the proteasome-inhibited cells, using conventional fluorescent microscopy indicated that only a fraction of the paraspeckle-associated *Neat1* and *Ctn RNA* displayed complete co-localization (Fig. 5Be–h). To achieve a better understanding of the localization of these RNA molecules in paraspeckles, we used Super-resolution structured illumination (SR-SIM) microscopy to determine the molecular organization of *Ctn RNA* and *Neat1* in MG132-treated and DRB-recovered transformed WT-MEFs (Fig. 6). We observed that under both conditions, *Ctn RNA* did not completely overlap with *Neat1* positive paraspeckles (Fig. 6B–E). This suggests that *Ctn RNA* decorated only a part of *Neat1*-nucleated paraspeckles (Fig. 6A–C). Furthermore, we observed that not all *Neat1*-nucleated paraspeckles contained *Ctn RNA* (Fig. 6A; see arrow). We further quantitated the ratio and degree of overlap of *Neat1* and *Ctn RNA* in paraspeckles (Fig. 6Da–c,Ea–c). The results showed that in DRB recovered cells, both *Neat1* and *Ctn RNA* foci display a more homogenous intraparaspeckle distribution and largely overlap with each other (Fig. 6Da–c). However, upon MG132 treatment, *Ctn RNA* and *Neat1* showed altered peak ratios and degree of overlap suggestive of a more heterogeneous intraparaspeckle distribution (Fig. 6Ea–c).

## Discussion

Previous studies suggest that sub-nuclear domains could be formed in two ways: (1) random self-organization or (2) ordered assembly^1^,^4^,^38^,^39^. Studies using a LacI reporter live cell imaging system have shown that paraspeckles do not organize by random self-organization^19^. The study by Mao *et al*.^4^ suggested that while PSPs within paraspeckles could facilitate the recruitment of other PSPs, they are unable to recruit RNA components to form *bona fide* paraspeckles. Instead, *Neat1* serves as the seeding molecule that recruits other components during the paraspeckle assembly^4^,^8^,^12^,^15^,^19^. Since paraspeckle components do not interact in a random manner, the interactions between these components merit investigation in order to understand how this sub-nuclear body assembles. While a number of studies have investigated the interaction of *Neat1* and PSPs, information on *Ctn RNA*, a paraspeckle-localized, non-structural RNA remains largely unavailable.

### Paraspeckles regulate the intranuclear organization of *Ctn RNA*

In ~30% of MEFs, a significant fraction of *Ctn RNA* forms discrete foci that localize to paraspeckles^26^. In contrast, upon disruption of paraspeckle structure, while *Ctn RNA* continues to form residual foci, a subset of *Ctn RNA* localizes to the perinucleolar space. This suggests that paraspeckles mediate the efficient compartmentalization of *Ctn RNA,* and in their absence *Ctn RNA* appears to acquire a more stochastic distribution in the nucleoplasm. Furthermore, in the absence of *Neat1*, *Ctn RNA* continues to associate with other PSPs, as indicated by RNA-FISH and RNA immunoprecipitation studies. This is in contrast to a previous study where the authors showed that structures induced by tethering of individual PSPs (instead of *Neat1*) to a LacI reporter construct did not retain *Ctn RNA*^19^. It is possible that artificial tethering of proteins may prevent interaction of these proteins with *Ctn RNA*, which might otherwise occur under physiological conditions.

### Paraspeckles do not impact nuclear retention of A-to-I edited *Ctn RNA*

Previous studies have suggested that paraspeckles are potentially involved in the nuclear retention of A-to-I edited transcripts in human cells^9^. For example, in undifferentiated human embryonic stem cells where intact paraspeckles are absent, in spite of robust A-to-I editing activity, the edited transcripts were not retained in the nucleus^9^. Furthermore, knockdown of *Neat1* and the consequent disruption of paraspeckle structure in HeLa cells resulted in the nucleocytoplasmic export of inverted Alu (IRAlu) containing mRNA^9^. In another study, it was documented that mRNAs with structured or edited 3′UTRs can be bound by a nuclear complex containing NonO, and such interaction prevents their export to the cytoplasm^40^,^41^. We observed that in the absence of intact paraspeckles (due to deletion of *Neat1*) or A-to-I editing (due to deletion of both ADAR1 & 2), *Ctn RNA* continued to localize in the nucleoplasm. Thus, in the case of *Ctn RNA*, *Neat1* or intact paraspeckles do not influence its nuclear retention in MEFs. We observed *Ctn RNA* positive residual nuclear foci in *Neat1* KO cells, some of which co-localized with other PSPs. At present, we cannot exclude the potential involvement of these residual nuclear foci in the nuclear retention of *Ctn RNA*. It is possible that association of *Ctn RNA* with other PSPs (such as SFPQ) in *Neat1* or NonO-depleted cells could facilitate the nuclear retention of Ctn RNA. Our results also demonstrate that A-to-I editing of *Ctn RNA* is not essential for its association with paraspeckles since *Adar1*/*Adar2* double KO MEFs showed paraspeckle localization of *Ctn RNA*.

### *Ctn RNA* foci are responsive to environmental triggers – transcriptional reactivation and proteasome inhibition

Previous studies have shown that transcriptional reactivation results in the reassembly of paraspeckles due to initiation of *Neat1* transcription^12^,^19^. In our study, we observe that *Ctn RNA* showed increased localization to paraspeckles upon transcriptional reactivation without a concomitant increase in *Ctn RNA* or *Neat1* levels. Earlier studies have demonstrated that paraspeckles tend to assemble in close proximity to the site of transcription of *Neat1*^4^,^10^,^19^. In contrast, *Ctn RNA* gene loci are located further away from paraspeckles as compared to *Neat1* transcription site^19^. Viewed in conjunction with our results, this suggests that after transcription, *Ctn RNA* localizes to paraspeckles possibly for its further processing or A-to-I editing. Future studies will investigate the potential involvement of paraspeckle in the processing of *Ctn RNA*.

In addition to being responsive to transcriptional reactivation, paraspeckles also show enlargement upon proteasome inhibition^23^. This enlargement is mainly due to the transcriptional upregulation of *Neat1*. The positive effect of proteasome inhibition on transcription has also been shown to occur in case of cyclooxygenase-2 (Cox-2) where increased level of this protein in response to proteasome inhibition has been attributed to its increased transcription^42^. Interestingly, in case of *Ctn RNA*, while we see only a marginal increase in total *Ctn RNA* levels upon proteasome treatment, we observe a significant increase in the size of *Ctn RNA* foci. At the ultrastructural level, as observed by SR-SIM, *Ctn RNA* adopts a more heterogeneous intraparaspeckle distribution in MG132-treated cells – with altered *Neat1: Ctn RNA* ratios and foci overlap. Therefore, *Ctn RNA* shows altered intra-paraspeckle organization upon proteasome inhibition^23^.

In summary, the results from this study further our knowledge about the organization and behavior of RNA components within the paraspeckle at several levels. First, non-structural RNA components of paraspeckles, namely, *Ctn RNA* forms residual foci in the absence of *Neat1*, though at a significantly lower level. Therefore, while *Ctn RNA* by itself forms ‘paraspeckle-like’ foci, *Neat1* improves the efficiency of foci formation. Second, *Ctn RNA* can associate with other paraspeckle components (PSPs) in the absence of *Neat1*. The efficiency of such interactions remains largely unaffected even in the presence or absence of intact paraspeckles. From this, we infer that the non-structural RNA components do not require an intact paraspeckle structure to associate with other PSPs. Lastly, RNA components of paraspeckles – both *Neat1* and *Ctn RNA* are responsive to environmental triggers, strengthening the view that paraspeckles function in response to certain stimuli.

## Materials and Methods

### Cell culture

*Adar1*/*Adar2*-KO MEFs^35^,^36^ were obtained from the Jantsch lab. Transformed WT-MEFs, *Neat1*-KO MEFs^24^ and *Adar1*/*Adar2*-KO MEFs were grown in DMEM containing high glucose, supplemented with penicillin-streptomycin and 10% foetal bovine serum (FBS) (HyClone, Logan, UT). For the 5,6-Dichloro-1-β-D-ribofuranosylbenzimidazole (DRB) recovery experiments, cells were treated with Ethanol or 25 μg/ml DRB (SIGMA, USA). For proteasome inhibition experiments, cells were treated with DMSO or 5 μM MG132 (SIGMA, USA).

### Reverse Transcription (RT), quantitative PCR, PCR

Total cellular RNA was isolated using Trizol (15596-018, Invitrogen, USA) according to the manufacturer’s instructions and reverse transcribed into cDNA using Superscript III First-Strand Synthesis System for RT-PCR (ThermoFisher Scientific, USA). qPCRs were performed using the Applied Biosystems StepOne Plus Real-Time PCR Systems (Applied Biosystems, USA). Transcript levels were quantitated against a standard curve by Real-Time RT-PCR using the SYBR Green fluorogenic dye and data analysed using the Applied Biosystems StepOne Plus Real-Time PCR Systems (Applied Biosystems, USA). Primer sets showing comparably high efficiencies were used for the analyses. The qPCR results were analysed using the comparative Ctmethod^43^.

### Transfection and siRNA/sh-RNA-mediated knockdown

*NonO* (L-048587-00-0005, 40 nM) (ON-TARGETplus smartpool siRNA, GE Dharmacon, USA) and *Adar1* siRNA (L-048587-00-0005, 150 nM) (ON-TARGETplus smartpool siRNA, GE Dharmacon, USA) were used to deplete *NonO* and *Adar1*, respectively. The siRNAs were transfected to cells using Lipofectamine RNAiMAX reagent as per the manufacturer’s instructions (Invitrogen, USA) and incubated for 48 hrs. Knockdown was confirmed using NonO antibody (gift from Dr Yasuyki Kurihara, Yokohama National University, Yokohama, Japan)^44^ and ADAR1 antibody (sc-73408; Santa Cruz Biotechnology, USA). Loading controls used were α-tubulin (T5168, SIGMA, USA) and B”-U2snRNP.

### Ribonucleoprotein Immunoprecipitation (RIP)

RIP was performed using an established protocol^45^. WT and *Neat1*-KO cells (1 × 10^7^) were used for RNA immunoprecipitation utilizing reversible chemical crosslinking of RNA-protein interactions by formaldehyde followed by immunoprecipitation using Anti-NonO antibody (9–99, gift from Dr. David Spector, CSHL, USA). Following IP, extracts were reverse cross-linked and total RNA was extracted using Trizol LS (Invitrogen, USA). Extracted RNA was treated with RNase-free DNase I (SIGMA, USA), and RT was conducted using random-hexamer primers as per the manufacturer’s instructions (Applied Biosystems, USA). qPCR was performed using gene-specific primers.

### Nuclear and cytoplasmic fractionation

Transformed WT-MEFs, *Neat1*-KO MEFs, control and *NonO* siRNA treated transformed WT-MEFs (1 × 10^6^) cells were used for fractionation. Cells were washed with PBS and re-suspended in RSB buffer (10 mM Tris-HCl pH7.4, 100 mM NaCl, 2.5 mM MgCl_2_, RNAse Inhibitor) and lysed in RSB buffer containing Digitonin (8 μg/ml) (SIGMA-ALDRICH, USA) for 10 min on ice. Cells were centrifuged (2000 rpm, 4 °C, 10 min) and the supernatant (cytoplasmic fraction) collected. The pellet (nuclear fraction) was washed with RSB and digitonin by the procedure described above. Trizol LS (10296-028, Invitrogen, USA) was added to the cytoplasmic fraction while Trizol was added to the nuclear fraction. Ct values of nuclear or cytoplasmic fractions were normalized to total RNA.

### RNA-FISH

To detect *Ctn RNA* and *Neat1*, RNA-FISH analysis was performed as previously described^45^. *Ctn RNA* localization to paraspeckles was increased during transcriptional reactivation. Therefore, for *Ctn RNA* FISH, cells were treated with the transcriptional inhibitor DRB followed by reactivation of transcription by removal of inhibitor with medium for 3 hrs. After RNA-FISH, immunofluorescence staining of NonO was performed using NonO antibody (1:100 for 2 hr at room temperature; sc-376865, Santa Cruz Biotechnology, USA) as previously described^45^. The paraspeckle number and percent co-localization was counted or measured respectively, by eye as the *Ctn RNA*/*Neat1*/*NonO* foci are very distinct and prominent. Unless indicated, hundred cells were counted in each experiment and the experiments were performed in biological replicate.

### Super-resolution structured illumination microscopy (SR-SIM) image acquisition and image processing

Images were acquired by SR-SIM ELYRA system with Axio Observer Z1 microscope from ZEISS. 3-color imaging was performed using 488 nm, 561 nm and 632 nm lasers. Exposure time was 100 ms or less for all three channels. Exposure time and excitation power was adjusted to maximize the signal without saturating the camera. SIM images were reconstructed using commercial softwares (ZEN 2012 and ZEN 2011 from Zeiss). Profile along the drawn line was created applying default setting in ZEN 2012.

All methods were carried out in accordance with relevant guidelines and regulations. The University of Illinois biosafety committee approved all the experimental protocols.

## Additional Information

**How to cite this article**: Anantharaman, A. *et al*. Paraspeckles modulate the intranuclear distribution of paraspeckle-associated *Ctn RNA.* *Sci. Rep.* **6**, 34043; doi: 10.1038/srep34043 (2016).

## Supplementary Material

Supplementary Information
