# Supplementary material for: Paraspeckles modulate the intranuclear distribution of paraspeckle-associated Ctn RNA
Source: Sci Rep. 2016 Sep 26;6:34043. doi: 10.1038/srep34043 (PMC5036046; doi:10.1038/srep34043)
Supplement: Supplementary Information [file srep34043-s1.pdf]

Supplementary Information

Paraspeckles modulate the intranuclear distribution of paraspeckle-associated *Ctn RNA*

Aparna Anantharaman[1], Mahdieh Jadaliha[1], Vidisha Tripathi[1#], Shinichi Nakagawa[2], Tetsuro Hirose[3], Michael F. Jantsch[4], Supriya G. Prasanth[1] and Kannanganattu V. Prasanth[1]

Supplementary Figures:

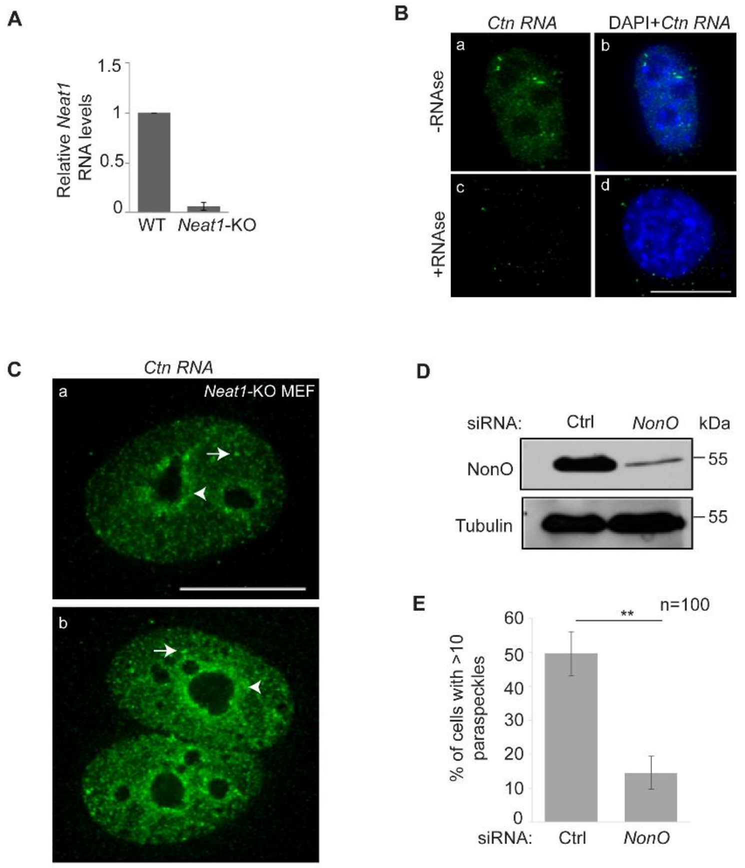

Figure S1. Controls to show that *Ctn RNA* is nuclear-retained in absence of *Neat1* and forms residual foci. (A) RT-qPCR analysis of total *Neat1* RNA levels in WT and *Neat1*-KO MEFs. (B) RNA-FISH analysis of *Ctn RNA* localization with or without RNAse treatment in WT-MEFs. Scale bar indicates 10 µm. (C) RNA-FISH analysis of *Ctn RNA* localization in *Neat1*-KO MEFs.

Scale bar indicates 10 µm. Arrow indicates *Ctn RNA* positive nuclear foci and arrowhead indicates the peri-nucleolar localization of *Ctn RNA* (D) Western blot measuring the protein levels of NonO in control and *NonO* siRNA-treated transformed WT-MEFs. Tubulin was used as loading control. (E) Graph showing the % of cells containing >10 parapspeckles/cell in control and *NonO* siRNA treated transformed WT-MEFs. n indicates number of cells that were counted. *Gapdh* was used as the normalization control in RT-qPCR experiments.

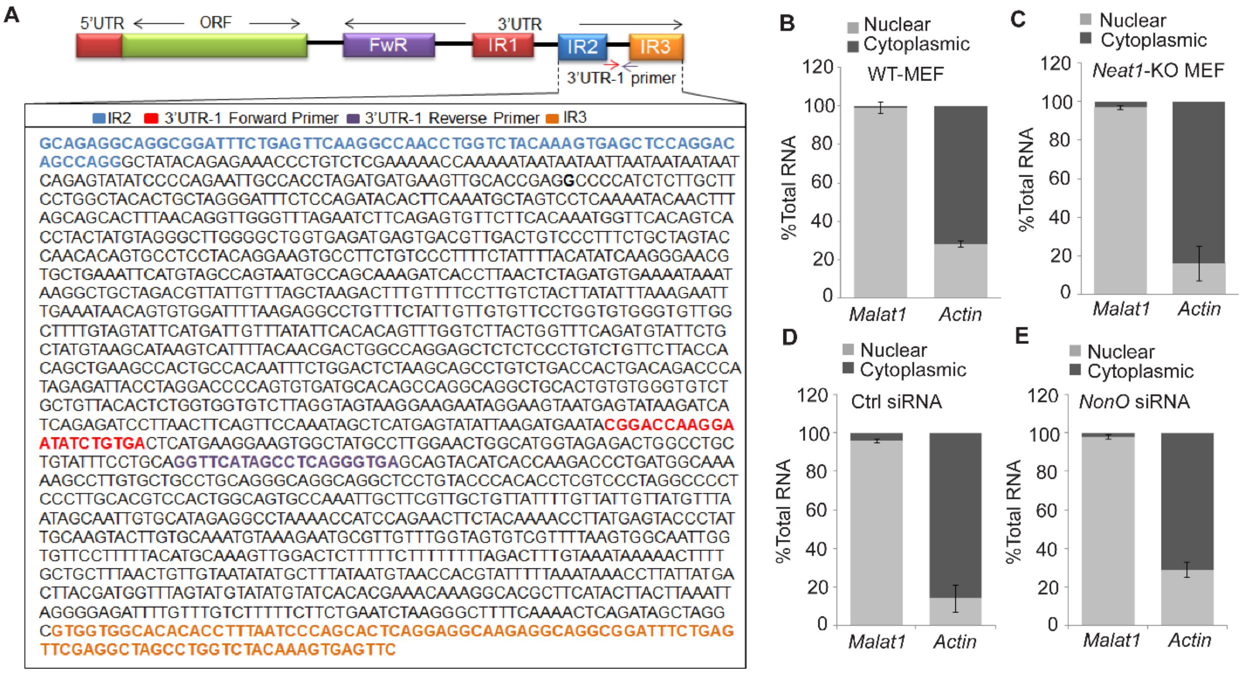

Figure S2. 3'UTR-1 primer location and markers for nuclear-cytoplasmic fractionation (A) Schematic showing the position of 3'UTR-1 primer pair. Please note that 3'UTR-1 primer pairs lies between inverted repeat 2 and 3. (B) RT-qPCR analysis of total levels of *Malat1* and *Actin* RNA in nuclear-cytoplasmic fractions of transformed WT-MEFs to measure the purity of nuclear (*Malat1*) and cytoplasmic (*Actin*) fractions. (C) RT-qPCR analysis of total levels of *Malat1* and *Actin* RNA in nuclear-cytoplasmic fractions of *Neat1*-KO MEFs to measure the purity of nuclear (*Malat1*) and cytoplasmic (*Actin*) fractions. (D) RT-qPCR analysis of total levels of *Malat1* and *Actin* RNA in nuclear-cytoplasmic fractions of control siRNA treated transformed WT-MEFs to measure the purity of nuclear (*Malat1*) and cytoplasmic (*Actin*) fractions. (E) RT-qPCR analysis of total levels of *Malat1* and *Actin* RNA in nuclear-cytoplasmic fractions of *NonO* siRNA treated transformed WT-MEFs to measure the purity of nuclear (*Malat1*) and cytoplasmic (*Actin*) fractions. *Gapdh* was used as the normalization control in RT-qPCR experiments.

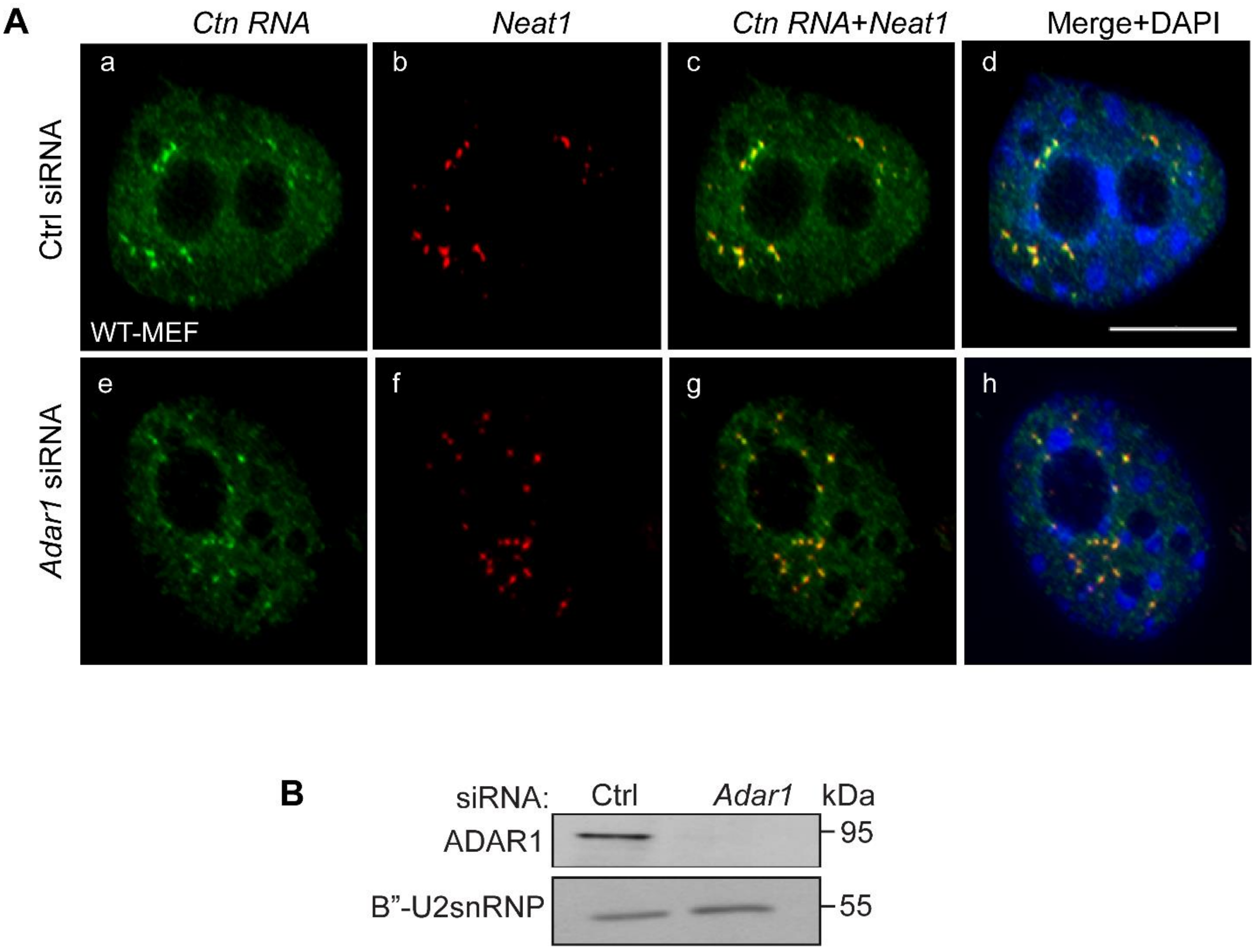

Figure S3. *Ctn RNA* is nuclear-retained in absence of ADAR1. RNA-FISH analysis of *Ctn RNA* and *Neat1* RNA localization in control and *Adar1*-depleted DRB-recovered transformed WT-MEFs. Scale bar indicates 10µm. (B) Western blot measuring the total protein levels of *Adar1* in control and *Adar1* siRNA-treated transformed WT-MEFs. B”-U2snRNP was used as loading control.

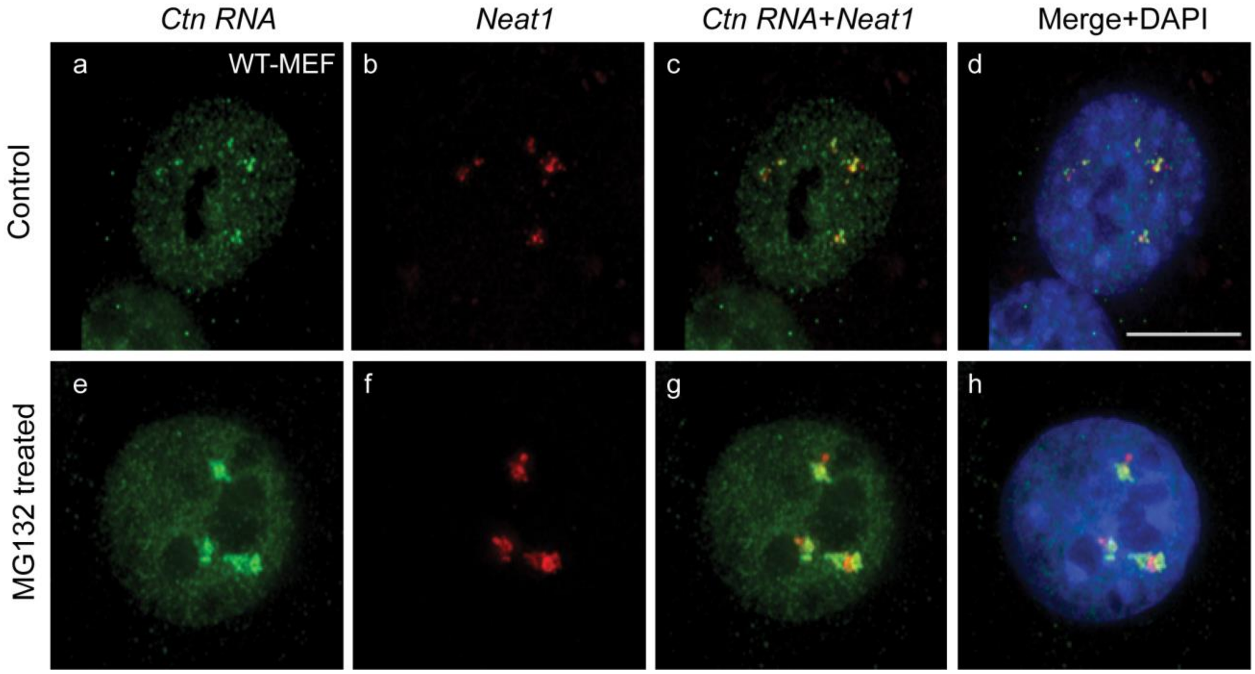

Figure S4. ***Ctn RNA*** **forms enlarged foci upon proteasome inhibition.** RNA-FISH analysis of *Ctn RNA* and *Neat1* localization in a single cell of control (DMSO-treated) and MG132-treated transformed WT-MEF.

Supplementary Table S1. Sequences of Primers used in this study

Primer Name	Primer Sequence
3’UTR-1	Forward Primer: 5’-CGGACCAAGGAATATCTGTGA-3’
	Reverse Primer: 5’- TCACCCTGAGGCTATGAACC-3’
3’UTR-2	Forward Primer: 5’-TCAGAAACACCCAGTCATGC-3’
	Reverse Primer: 5’-GGGCAACTAGCATTTGTGGT-3’
Neat1	Forward Primer: 5’-CTGGTTTATCCCAGCGTCAT-3’
	Reverse Primer: 5’-CTTACCAGACCGCTGACACA-3’
Actin	Forward Primer: 5’-CTCGTGTGGATCGGTGGCT-3’
	Reverse Primer: 5’-GCTGATCCACATCTGCTGGAA-3’
Gapdh	Forward Primer: 5’-CAGAGGCCCTATCCCAACTC-3’
	Reverse Primer: 5’-GGTCTGGGATGGAAATTGTG-3’
Malat1	Forward Primer: 5’-AGACCCTTCACCCCTCACCT-3’
	Reverse Primer: 5’-TTTTCTGGTGCAACCCACAG-3’
Malat1-2	Forward Primer: 5’-CGTTTGAAGGCATGAGTTGG-3’
	Reverse Primer: 5’-TGCCTCCCAAGTGCTAGGAT-3’
